# Genetic Parameters and Genotype × Diet Interaction for Body Weight Performance and Fat in Gilthead Seabream

**DOI:** 10.3390/ani13010180

**Published:** 2023-01-03

**Authors:** Stavroula Oikonomou, Zoi Kazlari, Dimitrios Loukovitis, Arkadios Dimitroglou, Lefteris Kottaras, Konstantinos Tzokas, Dimitrios Barkas, Nikolaos Katribouzas, Leonidas Papaharisis, Dimitrios Chatziplis

**Affiliations:** 1 Laboratory of Agrobiotechnology and Inspection of Agricultural Products, Department of Agricultural Technology, School of Geotechnical Sciences, International Hellenic University, Alexander Campus, P.O. Box 141, Sindos, 57400 Thessaloniki, Greece; 2 Research Institute of Animal Science, ELGO Demeter, Paralimni, 58100 Giannitsa, Greece; 3 Department of Animal Science, Agricultural University of Athens, Iera Odos 75, 11855 Athens, Greece; 4 Department of Research & Development, Avramar Aquaculture SA, 341 00 Chalkida, Greece

**Keywords:** gilthead seabream, plant proteins, genotype by diet interaction, heritability

## Abstract

**Simple Summary:**

The use of fishmeal and fish oil has long been an issue of major concern since nutrition for most aquaculture farms entails a considerable cost. To reduce the cost of the nutrition, many efforts have been made to replace the fishmeal/fish oil, either partially or totally, in diets by using plant-based proteins. The selected candidates per generation might be affected and the query of the establishment of a fish line selection used for a plant-based diet is under consideration. In the present study, fish were on different diets: a high-plant-protein diet containing 85% plant proteins and a high-animal-protein diet (standard commercial). During the experiment, fish were weighed and fat content was collected. We subsequently estimated the inheritance of the body weight performance and the fat content (0.39–0.85). Moreover, a genotype by diet interaction was re-evaluated causing a significant re-ranking of the selected candidates per diet. Furthermore, a higher genetic gain for body weight performance can be achieved by using the standard commercial diet, rather than the plant-based one. Thus, the establishment of a plant-based diet breeding line could be possible, if the reduction of the cost of aquafeed could balance the lower genetic gain expected to be achieved in each generation.

**Abstract:**

There has been thorough research on the genotype by diet interaction and the extent of its impact on the genetic evaluation, using a partly replaced marine animal protein on the gilthead seabream. To do that, 8356 individuals were gathered from two batches and followed different diets: a high-plant-protein diet containing 85% plant proteins and a standard commercial one containing 30% marine animal protein. During the experiment, body weight, growth and fat content were recorded. High heritability estimates were detected for the body weight performance and fat content. A small effect of genotype by diet interaction was detected in all phenotypes (presented as the genetic correlations from 0.95 to 0.97) but a medium-high ranking correlation between the breeding values for each trait was estimated (0.72–0.70). A higher expected response to selection for the body weight performance was detected using the standard commercial rather than the plant-based diet. Based on the findings, the establishment of a plant-based diet breeding strategy can be achieved provided the reduction of the cost of aquafeed is attained, though a lower genetic gain is expected.

## 1. Introduction

Human consumption of fish as an animal protein source has increased over the last decades because of the high nutrient quality of the fish. This consumption has to be covered by the aquaculture species since the capture fisheries are not able to meet the consumer demands and their quota in most countries has been stabilized over the last years [1]. A major concern for most aquaculture farms is that nutrition and the associated cost of the use of fish meal and fish oil is increasing; it is considered a necessity in an aquafeed. The availability of the last two raw materials is sometimes limited and their prices fluctuate, largely because of the increase in aquaculture production [2]. In order to reduce the cost of the nutrition, many efforts have been made to use alternative raw materials as a source of protein, such as plant-based proteins [1,2,3,4,5], some of these studies showed that a part of fishmeal and fish oil could be replaced in a fish diet [6]. Apart from the nutrition issues, the breeding programs that are running, sometimes on a large scale in aquaculture farming, have a target to improve the genetic gain for specific phenotypes per generation [7,8,9,10]. The main selection trait is body weight [8] and the average genetic gain for body weight that is observed is a 12.7% improvement per generation with a range of 2.3% to 42%, based on Gjedrem and Rye [9], who summarized the information from most of the studied species. In gilthead seabream, 7.3% and 22% is the expected genetic gain per generation for the body weight [9,11,12]. However, some phenotypes, especially for the quantitative traits such as body weight and growth, showed changes in performance in different environments, which indicates the existence of genotype interaction with the environment. In simple terms, not only is the phenotype affected by the environmental factors but also the performance of different genotypes changes. As a result, the ranking of the selection candidates in the genetic evaluation is fluctuating [7,10]. Thus, two major concerns for the partial or total replacement of the fishmeal/fish oil in diets for fish farming species for a breeding strategy are the potential re-ranking of the fish, which affects the selected candidates per generation, along with the query of the establishment of a fish line selection used for plant-based diet [13]. In some species, the replacement of the fishmeal/fish oil has been studied at the genetic level and the genotype by diet interaction has been investigated (*Oncorhynchus mykiss*, *Coregonus lavaretus*, *Ictalurus punctatus* and *Dicentrarchus labrax*, [13,14,15,16,17,18,19,20,21]. Some of those studied showed the presence of G X D [13,17,18,19,20,21], which affected the re-ranking of the families [13,17,18]. However, the establishment of a fish line selection used for a plant-based diet might not be needed (i.e., prediction of the genetic gain [20]). Focusing on the gilthead seabream, even though the replacement of the part or the total of fishmeal has been investigated [1,2,3,4,5,6], the existence of genotype by diet interaction has not been studied yet for the body weight and fat. So, the two main concerns of the re-ranking of the selection candidates within different selection lines per diet remain unanswered.

The aim of the study is first to investigate behavior of the estimated genetic parameters for the body weight, growth and fat under different protein source diets to identify any possible genotype × diet interaction using a plant-based diet in comparison with a standard commercial diet (standard diet using fishmeal/fish oil), to study any potential effects of possible genotype by diet interaction on the genetic evaluation and the selection decision on the broodstock candidates and finally to investigate the expected genetic gain on a plant-protein-based diet and on a fishmeal/fish-oil-based diet (standard diet).

## 2. Materials and Methods

### 2.1. Ethical Statement

All experiments were performed in accordance with relevant guidelines and regulations. Nireus S.A. research facilities are certified and licensed for the rearing and use of fish for scientific purposes (EL04-BIOexp-01). The animal study protocol was approved by the Departmental Animal Care Committee following the Three Rs principle, in accordance with Greek (PD 56/2013) and EU (Directive 63/2010) legislation on the care and use of experimental animals. All experimental procedures involving fish were supervised by FELASA accredited researchers.

### 2.2. Population

In the present study, 8356 gilthead seabream individuals from 216 full-sib families with a range of 14–48 offspring per family (only one family was represented by 1 offspring) were utilized. The fish originated from two reproductive periods (two batches) of a commercial company’s breeding program. Batch 16 was placed in the cages in July 2018 and batch 17 was placed in the cages in June 2019. In each batch, equal numbers of full-sib fish were placed in four different cages and were fed with two different diets (2 cages per diet). All fish were fed ad libitum (until saturation) two times per day. The experimental duration for batch 16 was more than 18 months, whereas batch 17 was more than 21 months. Sea water temperature fluctuated according to the seasonal changes from 15.2 to 28.0 °C for both experimental periods. Similarly, dissolved oxygen levels ranged from 5.0 to 8.4 mg L^−1^.

### 2.3. Diet Formulation

Fish from both batches (B16 and B17) were fed the same two diets. The first diet was a commercial-based diet based on marine raw materials and the second diet, or trial, had higher inclusions of plant-based raw materials (Table 1).

### 2.4. Data Collection

Body weight at tagging (W1) was measured at 130–169 days post hatching (DPH), the intermediate body weight (W2) was measured at 464–475 DPH and the final body weight (W3) was measured at 549–644 DPH. The fat content was recorded using a Distell fat meter (Distell FFM-692, Old Levenseat, Scotland, UK), for batch 16 at 549 DPH and for batch 17 at 644 DPH, by measuring 4 standard points on the same side for all fish and is expressed as the percentage of the body weight (FAT %). Growth 1 (G1 = W2 − W1) was estimated using the difference between intermediate body weight (W2) and body weight at tagging (W1), and growth 2 (G2 = W3 − W1) was estimated using the difference between the final body weight (W3) and body weight at tagging (W1).

### 2.5. Genetic Parameters

Heritability of body weight, growth and fat content, along with their genetic/phenotypic correlations, were estimated using the restricted estimation of maximum likelihood method (REML). The analyses were performed using AIREMLF90 [22]. Two alternative multivariate animal models were utilized, using the body weight at tagging (W1), intermediate body weight (W2), final body weight (W3) and fat content (FAT %), or the growth in initial period (G1), the growth in the total period in sea cages (G2), the final body weight (W3) and the fat content (FAT %) were used in order to estimate the genetic parameters among the studied traits. In these models, a fixed effect (with 2 levels) was used to describe the diet and another was added to describe the batch (with 2 levels), which can be depicted in the following model:

***Y* = *μ* + *a* + *b* + *Zu* + *e*** (1)

where ***Y*** corresponds to the matrix of W1, W2, W3 and fat, or G1, G2, W3 and fat content, ***μ*** is the mean of the above traits, ***a*** corresponds to the diet and cage, ***b*** is the batch, ***Z*** is the incidence matrix, ***u*** is the additive genetic effect utilizing the PRM and it is illustrated as ~N(0, Gσ_a_^2^) (G is the PRM and σ_a_^2^ is the additive variance). Finally, ***e*** is the residual.

### 2.6. Genotype by Diet Interaction

Firstly, the following linear model was used for each trait in order to identify the genotype by diet interaction (i.e., G × E interactions):

*Y* = *μ* + *a* + *b* + sire*a + dam*a + sire*dam + *sire* + *dam* + *e* (2)

where, ***Y*** corresponds to the vector of W2, W3, fat, G1 and G2 and, sire*a corresponds to the interaction between sire and diet, dam*a corresponds to the interaction between dam and diet, sire*dam corresponds to the interaction between dam and sire, ***sire*** and ***dam*** correspond to the parents fitted as random effect; the other parameters are the same as described above Equation (1). This analysis was performed in R [23].

Secondly, in order to estimate the genetic parameters and to quantify any possible genotype × diet, a bivariate animal model for each possible pair of traits was used. For each phenotype, each group of fish was considered as a different trait in the analysis in the bivariate model. In simple terms, the phenotypes of the fish that consumed the plant-based diet (i.e., environment 1) were considered as a trait whereas the fish that consumed the standard commercial diet (i.e., environment 2) were considered as another trait [10]. Consequently, for each trait, two heritability estimates (one for the plant diet and another for the standard commercial) were produced and the genetic correlation between them was estimated. One way to quantify any possible genotype × diet or environment interaction is the genetic correlation between the same traits measured under different diets fed, so the higher the genetic correlation, the smaller the G × E interaction becomes [24].

#### 2.6.1. Ranking Correlations

In order to quantify if any genotype by diet (G × D) interaction, causes any changes in the selection of candidates (i.e., selection decision), the ranking correlation between the estimated breeding values (EBVs) was estimated, since any changes in the ranking of the selection candidates (according to their EBVs) would affect such a selection decision. Therefore, the next step was to estimate the EBVs for each bivariate animal model in order to calculate the ranking (Spearman’s) correlation between EBVs estimated when the fish were fed the plant feed and their sibs the standard commercial feed and vice versa. The breeding values were estimated using BLUPF90 [22]. The ranking process included: ordering the fish based on their breeding values from the lowest to the highest ones and a serial number showing their position in a series based on the diet. This was used for each bivariate model and was repeated for W2, W3, G1, G2 and fat content. Then, a smaller group of fish (600) with the highest EBVs for each phenotype related to body weight performance (W2, W3, G1 and G2) and another group (600) with the lowest EBVs for fat were selected for the plant-based diet and for the standard commercial diet. Then, the ranking correlation (Spearman’s) coefficient between them was calculated. The descriptive statistics and the plots along with the correlation coefficient were calculated using R [23].

#### 2.6.2. Prediction of the Genetic Gain

The selected groups of the 600 fish with the higher EBVs for each phenotype related to growth (W2, W3, G1 and G2) and the lowest EBVs for fat were selected for the genetic evaluation model in order to estimate the effect of the changes on the selection decision due to selection candidates re-ranking for each trait. Then, the selection differential for each trait was estimated using the following formula S = S _average of the selected fish_ − S _average of the population_ [10]. Consequently, the selection differential was multiplied with the heritability estimate of each trait in order to calculate the expected response to selection (R = S h^2^) for each trait.

## 3. Results

### 3.1. Descriptive Statistics of the Population

Descriptive statistics of the population and per batch are illustrated in Table 2. The total number of the studied population was 8356 fish, from them, 4250 fish belong to batch 16 and 4106 fish to batch 17. The average tagging (W1), intermediate (W2) and final (W3) weight was 9.93 g, 321.38 g and 735.76 g, respectively. For G1 the average was 311.45 g and for G2 was 725.83 g. The average fat was 14.32% of the body weight.

### 3.2. Genetic Parameters

The heritability of the tagging, intermediate and final weight was 0.85, 0.42 and 0.39 respectively, whereas for fat content (%) it was 0.41 (Table 3 and Table 4). Moderate genetic correlation between W1 and W3, equal to 0.30, and between W1 and W2, equal to 0.46, were estimated. A high genetic correlation between final and intermediate weight was observed (0.90, Table 3). On the other hand, a moderate genetic correlation between FAT (%) and W2 or W3 was observed (0.34–0.35, Table 3). In Table 4, the heritability of G1 and G2 was 0.41 and 0.39, respectively. The genetic correlation of growth and W3 ranged from 0.90 to 0.99 (Table 4). The genetic correlation between the fat content and body weight or growth similarly ranged from 0.34 to 0.36. The genetic parameters per batch are illustrated in Supplementary Materials (Table S1, Table S2, Table S3 and Table S4). In batch 16, the heritability for W2 and W3 were higher than the estimation using the total population of both batches, whereas in batch 17, the heritability of W1 was lower than using the total population. For the fat content, the genetic correlation with the W1 was only 0.17 when analyzing only batch 17.

### 3.3. Genotype and Diet Interaction

Analyzing the body weight performance, the linear model revealed a significant interaction between the dam and diet; however, analyzing the fat content a significant interaction between sire and diet was found (Table 5). The fixed effects included in the same model, i.e., diet and the batch, also have a statistically significant effect on all traits (Table 5). The interaction between the sire and dam was significant in all the growth stages apart from fat content (Table 5).

Table 6 illustrates the heritability and the genetic correlation per phenotype, per diet and per batch from the various bivariate animal models. Small differences between the heritability estimates have been detected in all traits analyzing the full dataset (batch 16 and 17). On the other hand, analyzing only batch 16, higher differences in heritability estimates between the fish fed different diets, equal to 0.07–0.08, have been found for body weight and growth performance (W2, W3, G1 and G2). In the same group, fat content heritability followed the same pattern. In batch 17, a higher difference in heritability was identified in W3, G2 and fat compared with the heritability estimations for the total population.

Focusing on the genetic correlation for the body weight performance (W2, W3), growth and fat content under different diet regimes in the context of bivariate animal models, the range was from 0.95 to 0.97 for the total population (both batches), from 0.96 to 0.99 for batch 16 and from 0.90 to 0.95 for batch 17 (Table 6). The latter batch exhibited the lowest range of genetic correlation for the studied traits. The lowest genetic correlation was identified in fat content only in batch 17, whereas the highest was in W3 in batch 16. In practical terms, the genetic correlations for growth and body weight between fish fed different diets were very high (0.95–0.99, Table 6) suggesting that genetically the same trait is actually measured under different diets.

**Figure 1:**
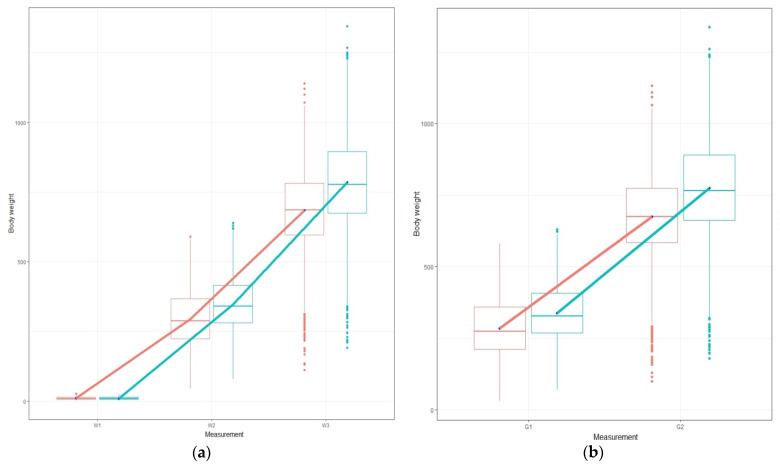
Plots of the body weight performance for the total population, (**a**) and (**b**) illustrate the body weight and the growth, respectively. The red line corresponds to plant-based diet and the blue line corresponds to standard commercial diet.

In Figure 1a,b, the body weight and growth for the total population per measurement and per diet is illustrated. In the same figure, the box plots per measurements per diet are also represented. In plots that illustrate the phenotype per diet, the parallel lines illustrate the absence of G × D interaction, whereas the non-parallel lines indicate the presence of it [7]. In our case, the lines from W2 to W3 (1a), as well as for growth (1b), are non-parallel, which indicates the existence of G × E but at a limited scale. Moreover, in Figure 1 all lines indicate diet sensitivity since higher population means appear for the standard commercial diet and lower means appear for the plant diet in all measurements [7]. The lines from W2 to W3, along with growth performance (G1 and G2), show heterogeneity of genetic variance on a limited scale, which is also validated based on the differences in the heritability estimations per diet [7].

The ranking (Spearman’s) correlations between the breeding values for each trait ranged from 0.72–0.70 using the total population, from 0.72 to 0.76 for batch 16 and from 0.66 to 0.69 for batch 17 (Table 7). Thus, the latter also showed the lowest range of genetic correlation and smaller differences between the estimated heritability (Table 6). The lowest ranking correlation coefficient between the EBVs was detected in fat in batch 17, whereas the highest was in W3 in batch 16 (Table 7).

Τhe ranking (Spearman’s) correlation coefficient between the EBVs for the final body weight of fish fed different diets was very low. Approximately 30% of the total fish will change ranking in their EBVs by using a different diet. Analyzing only batch 16, the ranking position of approximately 25% of the total fish will change by using a different diet, whereas in batch 17, the ranking position of approximately 32% of the total fish will change by using a different diet. Thus, the re-ranking of the EBVs is expected to vary in different years.

Nevertheless, following the applied selection intensity of the breeding program and selecting the 600 fish with the higher EBVs for body weight performance based on the plant diet, the ranking correlation between those EBVs and those estimated under the standard commercial diet ranged from 0.31 to 0.35. Selecting the same number of fish with the higher EBVs for body weight performance based on the standard commercial diet, the correlation between them and the plant diet ranged from 0.18 to 0.23. Similar effects were observed for the EBVs for fat content (Table 7). It seems that the effect of genotype by feed interaction becomes more apparent on the higher genetic merit selection candidates.

To sum up, the existence of the genotype by diet interaction in gilthead seabream was examined using three different approaches. The interaction, the multitrait analyses and the re-ranking of the fish (which can be performed using multitrait analyses based on the methods described in Sae-Lim et al. [7]). The plot was used but for visualization purposes. Based on the following findings, the statistically significant results from the interaction between diet and family (Table 5), the genetic correlation being less than unity (in our case ranges from 0.90–0.99 (Table 6)) and finally the reranking of the fish, which was 0.70–0.72 for the total population and 0.18–0.23 for the selected candidates, there is a genotype by diet interaction.

### 3.4. Response to Selection for Each Trait per Diet

Focusing on the expected response to selection for the body weight performance, higher genetic gain can be achieved using the standard commercial diet to genetically evaluate the selection candidates than using the plant-based diet. Especially for the intermediate and final body weight and the total growth, a difference from 8.83 to 20.3 gr per generation has been noted between the standard commercial and plant-based diets (Table 8). However, using the plant-based diet the fat content showed a higher decrease (0.18%) of the fat levels than the standard commercial diet (Table 8). It seems that evaluating the selection candidates using the standard commercial diet, a higher response to selection is expected for body weight and growth. On the contrary, that does not seem to be the case when the fat content is the selection objective.

## 4. Discussion

In the present study, using a substantial population of 8356 gilthead seabream selection candidates from a commercial breeding program and originating from two different reproductive years, full-sibs from each family were randomly divided into two sea cages each year and were fed on different diets: a high-plant-protein diet containing 85% plant proteins and a high-animal-protein diet containing 30% marine animal protein. These fish were used to estimate the genetic parameters for the body weight performance and fat content under different protein source diets, in order to: (a) investigate any possible genotype by diet interactions, (b) to study the effects of genotype by diet interaction on the genetic evaluation and (c) to investigate the expected genetic gain on a plant-protein-based diet and on a fishmeal/fish-oil-based diet (standard diet).

The heritability estimates using the multivariate animal model in the literature for gilthead seabream body weight at 130, 165 and 509 DPH were 0.28, 0.32 and 0.34, respectively [25]. At an older age, at 689–690 DPH the heritability estimates of the body weight were 0.25 or 0.29 [26,27]; similarly, at 980 DPH, the heritability estimate was 0.24 [28]. However, Gulzar et al. [29], estimated the heritability of the harvest weight (approximately 400 g) equal to 0.37 in a Greek farm (Galaxidi, Greece) and 0.55 in a farm in Spain. In our study, the heritability of the tagging weight at 130–169 DPH, was higher than the estimation of Navaro et al. [25]. However, our heritability decreased at each stage and in the final body weight (549–644 DPH) looks similar (0.39) to the heritability that is described by the aforementioned range and closer to the estimated heritability in the Greek farm in Galaxidi. When the analysis was performed for each batch separately, the heritability estimates for the body weight were slightly different between batches, higher heritability was estimated in batch 16 and lower in batch 17 (Table 5). The growth also followed same pattern: higher heritability in batch 16 than 17. As far as the fat content is concerned, different heritability estimates were reported for the heritability of the muscular fat (0.31, García-Celdrán et al.) [26] and for the fillet fat in a Greek site (0.46, Galaxidi) [29] and in a Spanish site (0.55, Cudomar) [29]. In our study the heritability estimate of fat content (0.41) was closer to the heritability estimates for fillet fat, which was estimated at the Galaxidi site.

Furthermore, several studies investigating the genotype × diet interaction have been made in other species, such as rainbow trout, European seabass, etc., using plant diets, revealing in general the presence of genotype × diet interaction using a plant protein diet [13,17,19,20,30]. However, no information has been found in the literature studying the genotype × diet interaction in gilthead seabream using a plant-based diet. In rainbow trout, Le Boucher et al. (2011) [19] studied the presence of genotype by diet interaction and reported significant interaction of sire and diet and of dam and diet for body weight at 343–465 days post fertilization (DPF). A significant family × diet interaction was detected in the body weight at the age of 18 weeks, whereas the same interaction was not significant for growth rate in rainbow trout [14]. In European sea bass, Le Boucher et al. [13] identified a significant sire x diet interaction. In our study, a significant interaction between dam and diet, as it was reported by Le Boucher et al. [19], and not between sire and diet was detected in body weight performance, as was reported by Le Boucher et al. [13,19] in European sea bass and in rainbow trout. Studying the fat content, a significant interaction was detected between sire and diet as it was detected in the last measurements of the fillet fat by Le Boucher et al. [13].

In rainbow trout, Le Boucher et al. [19] showed high genetic correlations (0.67–0.90) for body weight at different growth stages (213–464 DPF) indicating a G × D between the last three measurements of the body weight. In European sea bass, Le Boucher et al. [13] reported that the genetic correlation for the body weight was 0.51 to 0.81 (679–850 DPF), and for fillet lipid content was from 0.80 to 0.87 (736–787 rDPF). Also, Le Boucher et al. [20] also investigating the G × D, found that the genetic correlation of the body weight between diets ranged from 0.7 to 0.8 in European seabass. Focusing on the genetic correlations from the bivariate animal models in our study, they were high (0.90–0.99, Table 5) for each studied trait, illustrating that selecting candidates based on the standard commercial diet could lead to parallel genetic responses (Figure 1) in the plant diet [7]. Generally, when the genetic correlation between two traits is close to one, a possible pleiotropy and/or linkage among the genes affecting these traits is indicated [10] and consequently they may infer that the phenotypes are under the same genetic control in each environment (i.e., diet in our case). A threshold value for the genetic correlation, which can be used to consider whether an effect of G × E on each of the traits is significant or not, was suggested to be 0.8 [7,31]. For example, according to Robertson [31] a threshold value for genetic correlation of 0.8 between the same trait measurements under two different environments determines whether a different selection lines strategy (one for each environment) should be used in a breeding program. Based on that criterion, the effect of the G × E is considered “strong” and a two different lines strategy is required. Although such a threshold value could not indicate the statistical significance of the G × E, it is generally the “rule of thumb” in an applied breeding program. If the genetic correlation values are close to 0.8, it is necessary that a two breeding lines strategy is utilized. However, in such a scenario it is mandatory to perform a sib test with different diets, which should have a cost/benefit analysis. Such sib testing procedures have been used in the poultry breeding industry (D. Chatziplis, personal communications).

Nevertheless, Le Boucher et al. [13], when studying the European seabass, identified a strong G × D in the last measurements of the body weight; however, in this case a total replacement of the fish meal was used, whereas in our study, only a part of the fish meal was replaced. Possibly, the percentage of the replacement of the fishmeal could affect the presence of the G × D [18]. For the gilthead seabream, a significant G × D interaction has been found when studying the specific growth rate (growth divided per day) but with a weak effect using a linear mixed model and the phenotypic correlation [32]. In our study, however, we used the body weight at three different times and growth, as a difference between them, and the fat. Even though the effect of G × D is not very strong compared with the aforementioned threshold (0.8, [7,31]), the re-ranking of the selection candidates in a genetic evaluation is quite high. Palti et al. [15] reported that the ranking of the families between the diets was similar in rainbow trout, whereas in European sea bass, Le Boucher et al. [13] identified a significant re-ranking, following the genetic evaluation, of the sire families between diets for the body weight (605–850 DPF) and fillet lipid content (736–850 DPF). We notice a significant re-ranking of the fish using the bivariate animal model for the body weight, growth and fat content as was mentioned earlier. The re-ranking is further enhanced in the selected brooders after applying the same selection intensity in the selection candidates. However, the re-ranking of the selection candidates is quite extensive (0.18–0.33), which means that only 18–33% of the fish were in the same position independently of diet. This proportion could possibly justify the design of a breeding program based on the diet (i.e., a selection line for a plant-based diet and a selection line for the standard commercial diet) but it could not offer significant improvement of the breeding goal in gilthead seabream in the aquaculture farm. This can be observed since a higher genetic gain can be achieved by selecting while using the standard commercial diet instead of a plant diet in the terms of the body weight performance (based on the estimated genetic gain which was achieved per diet in the present study (Table 7)). The aforementioned findings agreed with Le Boucher et al. [20], in which higher genetic gain can be achieved for body weight using the control diet for 5% of selection intensity. However, a different pattern was observed in relation to the selection for fat content (%), where a higher genetic gain is expected using the plant diet; however, it should be noted that the average values for fat content were lower in the plant-based diet than the control. According to Sae-Lim et al. [7], a higher genetic gain can be achieved using high selection intensity when G × E exists, and indeed, in our study a high genetic gain was detected with selection intensities (1.26%); however, the ranking correlation coefficient between the plant and standard commercial diet was extremely low, which indicates a strong re-ranking of the fish (Table 7). Nevertheless, the establishment of a plant-based diet breeding strategy could be possible, if the reduction in the cost of aquafeed could balance the lower genetic gain that is expected to be achieved in each generation. However, it should be noted that this has to be re-evaluated when more selection objectives and more selection criteria are utilized in a breeding program. Alternatively, the cost effectiveness of a sib testing procedure with the alternative diet (e.g., plant-based diet) should be examined and reviewed before its incorporation.

Focusing on the selected methodology of the present study for the investigation of the genotype by diet interaction, firstly a linear model fitting the interaction effect Equation (2) was selected in order to identify the presence of G × D and more specifically the interaction between parents and diet, and secondly the bivariate animal model (using as trait 1 the phenotypes of the plant-based diet and as trait 2 the phenotypes of the standard commercial diet for each trait measured) was used in order to identify the effects of G × D in detail to estimate the existence of heterogeneity of variance and to investigate the re-ranking of the selection candidates for the studied traits [7]. According to Sae-Lim et al. [7], for a trait with moderate heritability approximately 10 fish per family and 200 families are needed to identify the G × E using the multitrait animal model. In our study, based on the heritability of the studied traits (which ranged between 0.39–0.42), there is a satisfactory representation of each family since the range of offspring was 12 to 48 offspring (apart from one family which included only one offspring) and 216 families were included in the present analysis. The optimal sample size is 2000 fish for a trait with moderate heritability; in our study, 8356 gilthead seabream fish from a commercial breeding program were used.

Even though two-year batches were included in the present analysis, no selection of candidates based only on the plant diet was performed in order to structure the families and to evaluate the theoretical genetic gain (Table 8) using two generations and a selection line for plant diet and standard commercial diet. Selecting candidates based on the plant diet could provide a validation of the overall estimations in the present analysis, since the present estimation of the genetic gain appears to be biased because fish were selected from many generations using the standard commercial diet.

## 5. Conclusions

In our study, the genotype by diet interaction for body weight performance and fat was studied for gilthead seabream. The effect of G × D is not very strong but the re-ranking of the selection candidates in a genetic evaluation is quite extensive (0.18–0.33), which means that only 18–33% of the fish were selected independently of the diet. This proportion could possibly justify the establishment of two selecting lines, one for the plant-based diet and another for the standard commercial diet, but it could not improve the production, since a higher genetic gain can be achieved by selecting using the standard commercial diet instead of a plant diet. However, the establishment of a plant-based diet breeding line could be possible as a scenario if the reduction of the cost of aquafeed could balance the lower genetic gain that is expected to be achieved in each generation. Sib testing procedures with the alternative diet within the breeding program could be an alternative cost-effective solution.

## Figures and Tables

**Table 1:** The raw materials and approximate composition (AOAC, 2003) of the standard commercial and plant-based diets. The comparative composition of the standard commercial diet and the plant-based diet is provided. Amino acids, fish oil and phospholipids were supplemented in both diets to meet the nutritional needs of gilthead sea bream.

Ingredients	Standard Commercial Diet	Plant-Based Diet
Fish meal standard %	24.6	
Fish meal LT %		8.1
Fish oil %	4.6	5.0
Salmon oil %	6.5	
Rapeseed oil %		6.5
Soya bean meal %	9.9	21.0
Rapeseed meal %		14.0
Soya protein concentrate %	17.0	7.0
Corn gluten %	14.5	9.6
Plant premix * %	6.6	8.0
Sunflower meal %	5.0	4.1
Wheat %	9.6	10.7
Amino acid premix ** %		3.2
Vitamin and mineral premix %	1.0	1.0
Hydrolyzed Lecithin source *** %	0.3	0.8
Ca & P source **** %	0.4	1.0
**Proximate analysis**	**Standard commercial diet**	**Plant-based diet**
Protein %	47.8	42.7
Lipids %	18.3	17.5
Ash %	6.8	5.9
Fiber %	2.9	4.5
Humidity %	7.6	8.7
Gross Energy (Mj/Kg)	21.42	20.64

* Mix of single-cell protein, by-products of amino-acids production and by-products of nGMO cereals’ fermentation; ** L-Lysine 79%, DL-Methionine 99% and L-Threonine 98,5%; *** FRA Lecimax; **** Monocalcium phosphate and carbon carbonate.

**Table 2:** Descriptive statistics of the population.

Population		W1 (g)	W2 (g)	W3 (g)	G1 (g)	G2 (g)	FAT (%)
**Total population**	Average ± SD	9.93 ± 4.86	321.38 ± 95.60	735.76 ± 161.63	311.45 ± 97.71	725.83 ± 163.45	14.32 ± 4.10
	Min	1.30	44.00	112.00	29.90	99.40	1.00
	Max	26.70	640.00	1345.00	630.20	1337.20	32.80
	Number of observations	8356	8353	8354	8353	8354	8356
**Batch 16**	Average ± SD	13.90 ± 3.38	260.56 ± 63.95	650.20 ± 118.71	246.66 ± 62.97	636.30 ± 118.08	16.28 ± 4.47
	Min	1.30	44.00	112.00	29.90	99.40	1.20
	Max	26.70	444.00	1000.00	430.90	986.20	32.80
	Number of observations	4250	4250	4248	4250	4248	4250
**Batch 17**	Average ± SD	5.82 ± 1.76	384.38 ± 81.04	824.29 ± 152.20	378.56 ± 80.48	818.46 ± 151.86	12.29 ± 2.33
	Min	1.60	59.00	135.00	54.50	130.10	1.00
	Max	15.00	640.00	1345.00	630.20	1337.20	20.10
	Number of observations	4106	4103	4106	4103	4106	4106

**Table 3:** Genetic parameters for body weight at different growth stages and fat content. Heritability is on the diagonal in bold; genetic and phenotypic correlations are above (in green) and below (in blue) the diagonal, respectively. Standard errors are illustrated in the parentheses.

	W1	W2	W3	FAT
**W1**	0.85 (0.05) *	0.46 (0.06) *	0.30 (0.07) *	−0.05 (0.08)
**W2**	0.34 (0.02) *	0.42 (0.04) *	0.90 (0.02) *	0.34 (0.07) *
**W3**	0.22 (0.02) *	0.82 (0.01) *	0.39 (0.04) *	0.35 (0.07) *
**FAT**	0.05 (0.02)	0.35 (0.02) *	0.43 (0.02) *	0.41 (0.04) *

* Significant estimation.

**Table 4:** Genetic parameters for growth at different stages and fat. Heritability is on the diagonal in bold; genetic and phenotypic correlations are above (in green) and below (in blue) the diagonal, respectively. Standard errors are illustrated in the parentheses.

	G1	G2	W3	FAT
**G1**	0.41 (0.04) *	0.90 (0.02) *	0.90 (0.02) *	0.35 (0.07) *
**G2**	0.82 (0.01) *	0.39 (0.04) *	0.99 (0.00) *	0.36 (0.07) *
**W3**	0.83 (0.01) *	0.99 (0.00) *	0.39 (0.04) *	0.35 (0.07)*
**FAT**	0.35 (0.02) *	0.43 (0.02) *	0.43 (0.02) *	0.41 (0.05) *

*Significant estimation.

**Table 5:** Level of significance (*p*-values) for the factors included in the linear model for interactions.

Phenotype	Batch	Diet	Dam	Sire	Sire*Dam	Dam*Diet	Sire*Diet
**W2**	<0.01	<0.001	0.672	0.733	0.011	<0.001	0.856
**W3**	<0.01	<0.001	0.761	0.778	0.04	0.019	0.90
**G1**	<0.01	<0.001	0.671	0.737	0.012	<0.001	0.852
**G2**	<0.01	<0.001	0.759	0.778	0.005	0.02	0.899
**FAT**	<0.01	<0.001	- ^1^	< 0.001	0.94	0.865	<0.001

^1^ the value was not estimated.

**Table 6:** Bivariate heritability and genetic correlation under different diets.

Population	Phenotype	Heritability		Genetic Correlation
		Plant-Based	Standard Commercial	
**Total population**	W2	0.41 (0.04)	0.43 (0.04)	0.96 (0.02)
	W3	0.40 (0.04)	0.41 (0.04)	0.97 (0.02)
	G1	0.40 (0.04)	0.42 (0.04)	0.95 (0.02)
	G2	0.40 (0.04)	0.40 (0.04)	0.96 (0.02)
	FAT	0.43 (0.04)	0.42 (0.04)	0.96 (0.02)
**Batch 16**	W2	0.44 (0.06)	0.52 (0.07)	0.97 (0.03)
	W3	0.43 (0.06)	0.50 (0.07)	0.99 (0.02)
	G1	0.43 (0.06)	0.50 (0.07)	0.96 (0.03)
	G2	0.43 (0.06)	0.50 (0.06)	0.98 (0.02)
	FAT	0.40 (0.06)	0.41 (0.06)	0.98 (0.03)
**Batch 17**	W2	0.40 (0.06)	0.39 (0.06)	0.95 (0.04)
	W3	0.39 (0.06)	0.36 (0.05)	0.95 (0.04)
	G1	0.40 (0.06)	0.39 (0.06)	0.95 (0.04)
	G2	0.39 (0.06)	0.35 (0.05)	0.95 (0.04)
	FAT	0.50 (0.07)	0.45 (0.06)	0.90 (0.04)

**Table 7:** Ranking (Spearman’s) correlation between the breeding values (EBV) for the studied traits under different diets for all the selection candidates and the selected 600 fish for broodstock.

Phenotype	Total Population	Batch 16	Batch 17	600 Fish Selected Based on the Standard Commercial Diet	600 Fish Selected Based on the Plant Diet
**W2**	0.71	0.74	0.69	0.23	0.33
**W3**	0.72	0.76	0.69	0.20	0.35
**G1**	0.71	0.73	0.68	0.21	0.31
**G2**	0.72	0.75	0.68	0.18	0.35
**FAT**	0.70	0.72	0.66	0.28	0.25

**Table 8:** Prediction of the response to selection for the body weight performance, growth and fat.

	W2		W3		G1		G2		FAT	
	Plant Based	Standard Commercial	Plant Based	Standard Commercial	Plant Based	Standard Commercial	Plant Based	Standard Commercial	Plant Based	Standard Commercial
**Average of the selected 600 fish**	420.27	436.19	895.03	940.01	411.37	427.55	885.92	930.85	10.63	10.94
**Selection differential**	98.89	114.81	159.27	204.25	99.92	116.1	160.09	205.02	−3.69	−3.38
Response to selection *	**40.54**	**49.37**	**63.71**	**83.74**	**39.97**	**48.77**	**64.04**	**82.01**	**−1.59**	**−1.41**

* Estimated using the heritability of each trait per diet in Table 4.

## Data Availability

The data presented in this study are available on request from the corresponding author. The data are not publicly available due to commercial restrictions.

## Supplementary Materials

The following supporting information can be downloaded at: https://www.mdpi.com/article/10.3390/ani13010180/s1, Table S1: Genetic parameters for body weight at different growth stages and fat content for batch 16. Heritability is on the diagonal in bold; genetic and phenotypic correlations are above (in green) and below (in blue) the diagonal, respectively. Standard errors are illustrated in the parentheses. Table S2: Genetic parameters for body weight, growth and fat content for batch 16. Heritability is on the diagonal in bold; genetic and phenotypic correlations are above (in green) and below (in blue) the diagonal, respectively. Standard errors are illustrated in the parentheses. Table S3: Genetic parameters for body weight at different growth stages and fat content for batch 17. Heritability is on the diagonal in bold; genetic and phenotypic correlations are above (in green) and below (in blue) the diagonal, respectively. Standard errors are illustrated in the parentheses. Table S4: Genetic parameters for body weight, growth and fat content for batch 17. Heritability is on the diagonal in bold; genetic and phenotypic correlations are above (in green) and below (in blue) the diagonal, respectively. Standard errors are illustrated in the parentheses.
