# Supplementary material for: Genetic Parameters and Genotype × Diet Interaction for Body Weight Performance and Fat in Gilthead Seabream
Source: Animals (Basel). 2023 Jan 3;13(1):180. doi: 10.3390/ani13010180 (PMC9817679; doi:10.3390/ani13010180)
Supplement: Supplementary file 1 [file animals-13-00180-s001.zip › animals-2059340-supplementary.pdf]

Supplementary material S1

Table S1: Genetic parameters for body weight at different growth stages and fat content for batch 16. Heritability is on the diagonal in bold; genetic and phenotypic correlations are above the diagonal (in green) and below (in blue), respectively. Standard errors are illustrated in the parentheses.

	**W1**	**W2**	**W3**	**FAT%**
**W1**	**0.86**	0.51	0.32	-0.10
**W2**	0.38	**0.48**	0.89	0.31
**W3**	0.23	0.82	**0.47**	0.31
**FAT%**	0.03	0.39	0.45	**0.39**

Table S2: Genetic parameters for body weight, growth and fat content for batch 16. Heritability is on the diagonal in bold; genetic and phenotypic correlations are above the diagonal (in green) and below (in blue), respectively. Standard errors are illustrated in the parentheses.

	**G1**	**G2**	**W3**	**FAT%**
**G1**	**0.46**	0.90	0.90	0.33
**G2**	0.82	**0.46**	0.99	0.32
**W3**	0.82	0.99	**0.47**	0.31
**FAT%**	0.40	0.45	0.45	**0.39**

Table S3: Genetic parameters for body weight at different growth stages and fat content for batch 17. Heritability is on the diagonal in bold; genetic and phenotypic correlations are above the diagonal (in green) and below (in blue), respectively. Standard errors are illustrated in the parentheses.

	**W1**	**W2**	**W3**	**FAT%**
**W1**	**0.72**	0.50	0.34	0.17
**W2**	0.39	**0.39**	0.90	0.46
**W3**	0.27	0.84	**0.36**	0.47
**FAT%**	0.14	0.37	0.48	**0.45**

Table S4: Genetic parameters for body weight, growth and fat content for batch 17. Heritability is on the diagonal in bold; genetic and phenotypic correlations are above the diagonal (in green) and below (in blue), respectively. Standard errors are illustrated in the parentheses.

	**G1**	**G2**	**W3**	**FAT%**
**G1**	**0.38**	0.90	0.90	0.46
**G2**	0.84	**0.36**	0.99	0.46
**W3**	0.84	0.99	**0.36**	0.47
**FAT%**	0.37	0.48	0.48	**0.45**

Supplementary material 1

Table S1: Genetic parameters for body weight at different growth stages and fat content for batch 16. Heritability is on the diagonal in bold; genetic and phenotypic correlations are above

the diagonal (in green) and below (in blue), respectively. Standard errors are illustrated in the parentheses.

	W1	W2	W3	FAT%
**W1**	**0.86**	0.51	0.32	-0.10
**W2**	0.38	**0.48**	0.89	0.31
**W3**	0.23	0.82	**0.47**	0.31
**FAT%**	0.03	0.39	0.45	**0.39**

Table S2: Genetic parameters for body weight, growth and fat content for batch 16. Heritability is on the diagonal in bold; genetic and phenotypic correlations are above the diagonal (in green) and below (in blue), respectively. Standard errors are illustrated in the parentheses.

	G1	G2	W3	FAT%
**G1**	**0.46**	0.90	0.90	0.33
**G2**	0.82	**0.46**	0.99	0.32
**W3**	0.82	0.99	**0.47**	0.31
**FAT%**	0.40	0.45	0.45	**0.39**

Table S3: Genetic parameters for body weight at different growth stages and fat content for batch 17. Heritability is on the diagonal in bold; genetic and phenotypic correlations are above the diagonal (in green) and below (in blue), respectively. Standard errors are illustrated in the parentheses.

	W1	W2	W3	FAT%
**W1**	**0.72**	0.50	0.34	0.17
**W2**	0.39	**0.39**	0.90	0.46
**W3**	0.27	0.84	**0.36**	0.47
**FAT%**	0.14	0.37	0.48	**0.45**

Table S4: Genetic parameters for body weight, growth and fat content for batch 17. Heritability is on the diagonal in bold; genetic and phenotypic correlations are above the diagonal (in green) and below (in blue), respectively. Standard errors are illustrated in the parentheses.

	G1	G2	W3	FAT%
**G1**	**0.38**	0.90	0.90	0.46
**G2**	0.84	**0.36**	0.99	0.46
**W3**	0.84	0.99	**0.36**	0.47
**FAT%**	0.37	0.48	0.48	**0.45**
